# Coronary heart disease and risk factors as predictors of trajectories of psychological distress from midlife to old age

**DOI:** 10.1136/heartjnl-2016-310207

**Published:** 2016-11-18

**Authors:** Marianna Virtanen, Marko Elovainio, Kim Josefsson, G David Batty, Archana Singh-Manoux, Mika Kivimäki

**Affiliations:** 1 Finnish Institute of Occupational Health, Helsinki, Finland; 2 National Institute for Health and Welfare, Helsinki, Finland; 3 Institute of Behavioral Sciences, University of Helsinki, Finland; 4 Department of Epidemiology and Public Health, University College London, London, UK; 5 Inserm U1018, Centre for Research in Epidemiology and Population Health, Villejuif, France; 6 Clinicum, Faculty of Medicine, University of Helsinki, Helsinki, Finland

## Abstract

**Objective:**

To examine coronary heart disease (CHD) and its risk factors as predictors of long-term trajectories of psychological distress from midlife to old age.

**Methods:**

In the Whitehall II cohort study, 6890 participants (4814 men, 2076 women; mean age 49.5 years) had up to seven repeat assessments of psychological distress over 21 years (mean follow-up 19 years). CHD and its risk factors (lifestyle-related risk factors, diabetes, hypertension and cholesterol) were assessed at baseline. Group-based trajectory modelling was used to identify clusters of individuals with a similar pattern of psychological distress over time.

**Results:**

We identified four trajectories of psychological distress over the follow-up: ‘persistently low’ (69% of the participants), ‘persistently intermediate’ (13%), ‘intermediate to low’ (12%) and ‘persistently high’ (7%). The corresponding proportions were 60%, 16%, 13% and 11% among participants with CHD; 63%, 15%, 12% and 10% among smokers and 63%, 16%, 12% and 10% among obese participants. In multivariable adjusted multinomial regression analyses comparing other trajectories to persistently low trajectory, prevalent CHD was associated with intermediate to low (OR 1.70, 95% CI 1.08 to 2.68) and persistently high (OR 1.92, 95% CI 1.16 to 3.19) trajectories. Smoking (OR 1.33, 95% CI 1.07 to 1.64; OR 1.55, 95% CI 1.19 to 2.04) and obesity (OR 1.33, 95% CI 1.04 to 1.70; OR 1.47, 95% CI 1.07 to 2.01) were associated with persistently intermediate and persistently high trajectories, respectively.

**Conclusion:**

CHD, smoking and obesity may have a role in the development of long-lasting psychological distress from midlife to old age.

## Introduction

Depression is a major public health issue characterised by high lifetime onset, persistence over time and recurrence.^1^ Depression as a risk factor for incident coronary heart disease (CHD) has been extensively investigated,^2^ and high prevalence and incidence of depression in cardiac patients has been observed repeatedly.^3–5^ For the latter, depression might be a component of adjustment disorder due to a severe heart condition encountered although symptoms might be transient in nature.^3^ Alternatively, the ‘vascular depression hypothesis’ postulates that vascular pathology itself, that is, CHD and its risk factors, such as hypertension and hypercholesterolaemia, might predispose people to depression in later life.^5^ ^6^ Recently, subclinical common mental disorder, defined for example, as the presence of psychological distress, has also received attention because of its high prevalence in general populations and associations with future clinical depression, morbidity and mortality.^7^ ^8^

Although there is evidence that CHD in particular and, to a lesser extent, its risk factors might increase the risk of depressive symptoms in the short term,^5^ their contribution to long-term trajectories of symptoms has rarely been examined and the findings are inconsistent.^9^ In one study of older women, self-reported myocardial infarction (MI) and hypertension predicted increasing and persistently high symptom trajectories,^10^ while another study of older men and women did not find such associations.^11^

In this study, we sought to expand the existing knowledge on CHD and mental health by focusing on psychological distress, which includes symptoms of depression and anxiety, corresponding to a non-specific multimorbidity condition common in older adults.^12^ The analyses reported here had two purposes. Using seven assessments of psychological distress over a 21-year follow-up period, we first characterised distinct trajectories of psychological distress from midlife to old age. We then examined the extent to which CHD and its risk factors predicted these trajectories.

## Methods

### Participants and study design

The Whitehall II study is a prospective cohort study of British government employees initially including all London-based office staff, aged 35–55 years, who were working in 20 departments at study entry, 1985–1988.^13^ With a response rate of 73%, there were 10 308 participants in the baseline examination. Since this first wave of data collection, self-completion questionnaires and clinical data have been collected from study members every 2–5 years. We used data from waves 1 to 11, the last wave in 2012–2013 (excluding 4th and 10th due to lacking measurements of psychological distress). After complete description of the study to the participants, their written consent was obtained. Ethical approval for the Whitehall II study was obtained from the University College London Medical School and the NHS London-Harrow Health Research Authority committees on the ethics of human research. Whitehall II data, protocols and other metadata are available to bona fide researchers for research purposes (http://www.ucl.ac.uk/whitehallII/data-sharing).

Of the 10 308 participants at wave 1, 7575 participated and responded to questions on psychological distress at the baseline assessment of this study (wave 3, 1991–1994) and at least one other assessment during the subsequent six study waves at which psychological distress was measured. Of them, 6890 (4814 men, 2076 women; mean age 49.5 (SD 6.0) years) provided data on all covariates. The mean number of psychological distress measurements was 6.0 (SD 1.5, range 2–7) and the mean length of follow-up was 18.6 (SD 3.8) years. By the end of follow-up (31 August 2012), 557 participants (8.1%) died. They had an average of 4.0 (SD 1.4) measurements of psychological distress at follow-up.

### Measurement of psychological distress

At each examinations of the Whitehall II study (except the 4th and 10th), participants responded to the self-administered 30-item General Health Questionnaire (GHQ).^14^ The GHQ is a screening instrument designed to detect common mental disorder, such as depression and anxiety, and it has been shown to have high sensitivity (78%, 86%) and specificity (83%, 87%) for common mental disorder and depression, respectively, in the present cohort.^15^ Of the 30 items, 13 assess symptoms of depression (eg, ‘been thinking of yourself as a worthless person’), 9 assess problems in social function (eg, ‘been finding it easy to get on with other people’—reverse coding), 6 assess anxiety (eg, ‘been getting scared or panicky for no good reason’) and two assess sleep disturbances (eg, ‘lost much sleep over worry’). Each questionnaire item denotes a specific symptom; response categories are scored as either 1 or 0 to indicate the presence of that symptom. A total score of 5 or more signals ‘caseness’.^15^ History of psychological distress was defined as a caseness of GHQ in either wave 1 or wave 2 (1985–1990). The 21-year trajectories were assessed at seven study waves in which GHQ was assessed, beginning from wave 3 (1991–1994) and ending up to wave 11 (2012–2013).

### Assessment of prevalent CHD

We ascertained the baseline CHD using data from the three first study waves (1985–1988 to 1991–1994). Non-fatal CHD included non-fatal MI or definite angina, defined according to the MONICA criteria,^16^ based on study ECG, hospital acute ECGs and cardiac enzymes. Angina was defined on the basis of clinical records and nitrate medication use, excluding cases based solely on self-reported data (the Rose Questionnaire)^17^ without clinical verification. Classification was carried out independently by two trained coders, with adjudication for any disagreements.

### Measurement of CHD risk factors

We used data on CHD risk factors from the wave 3 (1991–1994) clinical examination. Smoking (yes/no) was based on survey questionnaire. Height was measured to the nearest 0.5 cm. Weight was measured to the nearest 0.1 kg. From these, body mass index (BMI) was measured and obesity was defined as a BMI ≥30 kg/m^2^. Weekly alcohol use from survey questionnaire was used to categorise alcohol consumption as non-use, alcohol use within recommended limits (women: up to 14 drinks/week; men: up to 21 drinks/week) and alcohol use above recommended limits (women: >14 drinks/week; men: >21 drinks/week).^18^ Systolic and diastolic blood pressure was measured twice while seated after a 5 min rest, and means for the two measurements for systolic and diastolic blood pressure were used in the analyses. Information on antihypertensive medication was collected from survey responses. Hypertension was defined as either having systolic blood pressure ≥140 mm Hg, diastolic blood pressure ≥90 mm Hg or as use of antihypertensive medication. Total cholesterol was assessed from blood serum samples, and hypercholesterolaemia was defined as total cholesterol ≥5.0 mmol/L or use of lipid-lowering drugs. To see whether the association between cholesterol level and trajectories of psychological distress is U shaped,^19^ an alternative three-class formulation was computed: <5.0, 5.0–<6.5 and ≥6.5 mmol/L (excluding the 52 participants with lipid-lowering drugs). Diabetes was defined as a fasting glucose ≥7.0 mmol/L or a 2-hour postload glucose ≥11.1 mmol/L on the oral glucose tolerance test performed during the Whitehall study clinical screening or as physician-diagnosed diabetes or use of diabetes medication.^20^

### Sociodemographic factors

Age, sex and socioeconomic status were based on the survey questionnaire at wave 3 (if socioeconomic status was missing, it was derived from previous waves). Socioeconomic status was based on the civil service occupational grades. Employment grade in the Whitehall II study is a comprehensive marker of socioeconomic position and is related to salary, social status and level of responsibility at work.^13^ The civil service identifies 12 grades that comprise clerical assistant, clerical officer, executive officer, higher executive officer, senior executive officer and seven ‘unified grades’. These were further grouped to the three categories: low, intermediate and high socioeconomic status.^13^

### Statistical analysis

Trajectories of psychological distress were defined using group-based trajectory models (GBTM), which identify clusters of individuals (trajectory groups) with a similar trajectory over time.^21^ GBTM is increasingly being applied to clinical research to map the developmental course of disease and to identify the number, shape and size of different (latent) trajectory groups in the data. We used the Bayesian Information Criteria and Akaike's information criterion to determine the optimal number of trajectories: lower absolute values correspond to better fit.^21^ We hypothesised a priori that there would be three to four latent trajectories, as suggested by other research on this topic.^9^

We applied multinomial regression analysis and expressed the results as ORs and their 95% CIs, where all trajectory groups that we found were analysed together and persistently low was set as an outcome reference group against which the association with CHD and its risk factors were evaluated. The estimates were adjusted for age, sex and socioeconomic status. The full model also included CHD risk factors (smoking, high alcohol use, obesity, hypertension, hyperlipidaemia, diabetes and history of psychological distress) and a sensitivity analysis for the level of psychological distress at wave 3 (with four categories: 0, 1–5, 6–10 and >10 points). GBTM was performed using Stata V.13 and all other analyses using SAS V.9.4.

## Results

**Figure 1:**
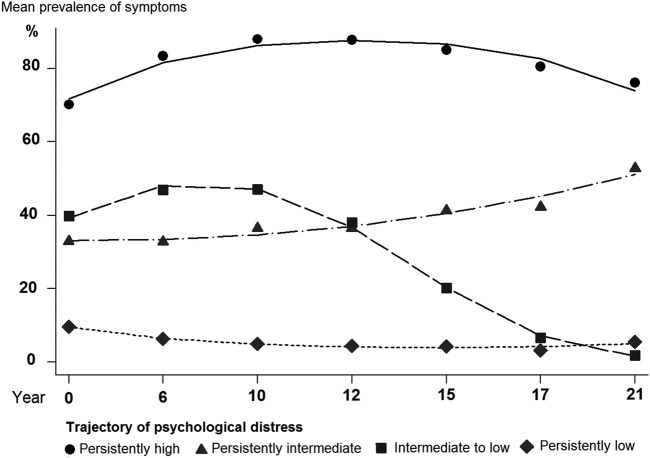
Trajectories of psychological distress symptoms (General Health Questionnaire-30) among the Whitehall II study participants.

A four-trajectory solution with all trajectories non-linear yielded the best fit (see online supplementary table S1). Figure 1 presents the shape of each trajectory: predicted probabilities of group membership totalled 69% with persistently low, 13% with persistently intermediate, 12% with intermediate to low and 7% with persistently high symptom trajectory. The corresponding proportions were 60%, 16%, 13% and 11% among participants with CHD; 63%, 15%, 12% and 10% among smokers and 63%, 16%, 12% and 10% among obese participants.

10.1136/heartjnl-2016-310207.supp1 Supplementary table 1

Descriptive characteristics of the participants in table 1 by their membership of a psychological distress trajectory suggest that older participants, men and those with high socioeconomic status, non-smoking habit, normal weight and no CHD were overrepresented in the persistently low trajectory.

**Table 1:** Descriptive characteristics of the participants at baseline by trajectories of psychological distress

	Trajectories				
	All (N=6890), n (%)	‘Persistently low’ (N=4731), n (%)	‘Persistently intermediate’ (N=882), n (%)	‘Intermediate to low’ (N=807), n (%)	‘Persistently high’ (N=470), n (%)
Age (years)					
<50	3721 (54.0)	2376 (50.2)	477 (54.1)	573 (71.0)	295 (62.7)
≥50	3169 (45.9)	2355 (49.8)	405 (45.9)	234 (29.0)	175 (37.2)
Sex					
Male	4814 (69.9)	3447 (72.9)	572 (64.9)	521 (64.6)	274 (58.3)
Female	2076 (30.1)	1284 (27.1)	310 (35.2)	286 (35.4)	196 (41.7)
Socioeconomic status					
High	2730 (39.6)	1933 (40.9)	343 (38.9)	314 (38.9)	140 (29.8)
Intermediate	3117 (45.2)	2096 (44.3)	398 (45.1)	389 (48.2)	234 (49.8)
Low	1043 (15.1)	702 (14.8)	141 (16.0)	104 (12.9)	96 (20.4)
Smoking					
No	6023 (87.4)	4189 (88.5)	750 (85.0)	699 (86.6)	385 (81.9)
Yes	867 (12.6)	542 (11.5)	132 (15.0)	108 (13.4)	85 (18.1)
Alcohol use					
No	1274 (18.5)	855 (18.1)	176 (20.0)	133 (16.5)	110 (23.4)
Within recommended limits	4525 (65.7)	3147 (66.5)	558 (63.3)	534 (66.2)	286 (60.9)
Above recommended limits	1091 (15.8)	729 (15.4)	148 (16.8)	140 (17.4)	74 (15.7)
Obesity					
No	6257 (90.8)	4335 (91.6)	780 (88.4)	734 (91.0)	408 (86.8)
Yes	633 (9.2)	396 (8.4)	102 (11.6)	73 (9.1)	62 (13.2)
Hypertension					
No	5488 (79.7)	3741 (79.1)	699 (79.3)	669 (82.9)	379 (80.6)
Yes	1402 (20.4)	990 (20.9)	183 (20.8)	138 (17.1)	91 (19.4)
High total cholesterol (≥5.0 mmol/L)					
No	601 (8.7)	402 (8.5)	66 (7.5)	88 (10.9)	45 (9.6)
Yes	6289 (91.3)	4329 (91.5)	816 (92.5)	719 (89.1)	425 (90.4)
Total cholesterol level (mmol/L)					
<5.0	601 (8.8)	402 (8.6)	66 (7.6)	88 (11.0)	45 (9.7)
5.0–<6.5	3050 (44.6)	2066 (44.0)	380 (43.5)	381 (47.6)	223 (48.0)
≥6.5	3187 (46.6)	2232 (47.5)	427 (48.9)	331 (41.4)	197 (42.4)
Diabetes					
No	6701 (97.3)	4597 (97.2)	857 (97.2)	786 (97.4)	461 (98.1)
Yes	183 (2.7)	134 (2.8)	25 (2.8)	21 (2.6)	9 (1.9)
Coronary heart disease					
No	6689 (97.1)	4610 (97.4)	850 (96.4)	781 (96.8)	448 (95.3)
Yes	201 (2.9)	121 (2.6)	32 (3.6)	26 (3.2)	22 (4.7)

In table 2, we show the age sex and socioeconomic status adjusted associations from multinomial regression analyses of baseline sociodemographic characteristics and CHD risk factors with the psychological distress trajectories, setting persistently low as a reference group. Female sex, smoking and obesity were associated with the likelihood of being on persistently intermediate symptom trajectory; younger age and female sex were associated with intermediate to low trajectory and younger age, female sex, intermediate and low socioeconomic status, smoking and obesity were associated with persistently high symptom trajectory. The corresponding analyses in which all risk factors, baseline CHD and history of psychological distress were mutually adjusted, are presented in online supplementary table S2, with no remarkable changes in the estimates. Further adjustment for the level of psychological distress at baseline (wave 3) provided the following estimates: for smoking persistently intermediate OR=1.32 (95% CI 1.06 to 1.65), intermediate to low OR=1.14 (95% CI 0.89 to 1.46) and persistently high OR=1.49 (95% CI 1.10 to 2.01); for obesity persistently intermediate OR=1.31 (95% CI 1.02 to 1.69), intermediate to low OR=1.07 (95% CI 0.80 to 1.43) and persistently high OR=1.47 (95% CI 1.04 to 2.08).

10.1136/heartjnl-2016-310207.supp2 Supplementary table 2

**Table 2:** Multinomial logistic regression analysis for the associations of sociodemographic factors and cardiovascular risk factors at baseline with trajectories of psychological distress

	‘Persistently intermediate’ versus ‘persistently low’	‘Intermediate to low’ versus ‘persistently low’	‘Persistently high’ versus ‘persistently low’
	OR (95% CI)*	OR (95% CI)*	OR (95% CI)*
Age (years)			
<50	1.00	1.00	1.00
≥50	0.90 (0.77 to 1.07)	0.41 (0.33 to 0.50)	0.55 (0.43 to 0.70)
Sex			
Male	1.00	1.00	1.00
Female	1.53 (1.29 to 1.81)	1.69 (1.42 to 2.01)	1.80 (1.45 to 2.24)
Socioeconomic status			
High	1.00	1.00	1.00
Intermediate	0.98 (0.83 to 1.15)	0.99 (0.84 to 1.17)	1.34 (1.07 to 1.67)
Low	0.90 (0.71 to 1.15)	0.78 (0.60 to 1.01)	1.47 (1.08 to 2.00)
Smoking			
No	1.00	1.00	1.00
Yes	1.34 (1.09 to 1.65)	1.16 (0.93 to 1.46)	1.55 (1.20 to 2.01)
Alcohol use			
Within recommended limits	1.00	1.00	1.00
No use	1.11 (0.91 to 1.35)	0.92 (0.75 to 1.14)	1.22 (0.96 to 1.56)
Above recommended limits	1.16 (0.95 to 1.42)	1.08 (0.88 to 1.33)	1.16 (0.88 to 1.52)
Obesity			
No	1.00	1.00	1.00
Yes	1.35 (1.07 to 1.71)	1.07 (0.82 to 1.40)	1.49 (1.11 to 1.99)
Hypertension			
No	1.00	1.00	1.00
Yes	1.06 (0.89 to 1.27)	0.97 (0.79 to 1.18)	1.06 (0.83 to 1.36)
High total cholesterol (≥5.0 mmol/L)			
No	1.00	1.00	1.00
Yes	1.23 (0.93 to 1.62)	0.98 (0.76 to 1.26)	1.05 (0.76 to 1.46)
Total cholesterol level (mmol/L)			
<5.0	1.00	1.00	1.00
5.0–<6.5	1.18 (0.89 to 1.56)	1.00 (0.77 to 1.29)	1.10 (0.78 to 1.55)
≥6.5	1.28 (0.96 to 1.70)	0.95 (0.72 to 1.23)	0.99 (0.70 to 1.41)
Diabetes			
No	1.00	1.00	1.00
Yes	1.07 (0.69 to 1.65)	1.16 (0.72 to 1.86)	0.73 (0.37 to 1.46)

*Adjusted for age, sex and socioeconomic status.

Table 3 shows the association between CHD and trajectories of psychological distress. In analysis adjusted for sociodemographic factors and persistently low trajectory as a reference group, CHD was associated with ending up on persistently intermediate (OR=1.62, 95% CI 1.08 to 2.41), intermediate to low (OR=1.92, 95% CI 1.24 to 2.99) and persistently high trajectory (OR=2.41, 95% CI 1.49 to 3.87). After multivariable analysis in model 2, the significant associations with intermediate to low and persistently high were attenuated but remained significant while the association with persistently intermediate became non-significant. We further adjusted the models with the level of wave 3 psychological distress (OR=1.34, 95% CI 0.88 to 2.05 for persistently intermediate; OR=1.67, 95% CI 1.03 to 2.71 for intermediate to low and OR=1.80, 95% CI 1.02 to 3.17 for persistently high, not shown in the table). We also found that there was no difference in the proportion of participants with missing data between trajectory groups (range 9%–11% with missing data p=0.113). We also performed the age, sex and socioeconomic status adjusted analyses (which were all non-missing variables) among participants with missing data on other covariates (n=7575) and found that the results for CHD, smoking and obesity were unchanged (not shown in the tables).

**Table 3:** Multinomial logistic regression analysis for the association between coronary heart disease (CHD) at baseline and trajectories of psychological distress

CHD at baseline	‘Persistently intermediate’ versus ‘persistently low’	‘Intermediate to low’ versus ‘persistently low’	‘Persistently high’ versus ‘persistently low’
Model 1*			
No CHD	1.00	1.00	1.00
CHD	1.62 (1.08 to 2.41)	1.92 (1.24 to 2.99)	2.41 (1.49 to 3.87)
Model 2†			
No CHD	1.00	1.00	1.00
CHD	1.38 (0.91 to 2.09)	1.70 (1.08 to 2.68)	1.92 (1.16 to 3.19)

*Adjusted for age, sex and socioeconomic status.

†Adjusted for age, sex, socioeconomic status, smoking, obesity, alcohol use, hypertension, cholesterol, diabetes and history of psychological distress.

## Discussion

In this study of middle-aged men and women with up to seven repeated measurements of psychological distress over a maximum of 21 years, we identified four distinct trajectories of psychological distress: persistently low (the most common trajectory; 69% of the participants), persistently intermediate (13%), intermediate to low (12%) and persistently high (7%) trajectories. These distributions are in line with previous studies which have found persistently low-symptom trajectories to be most common in midlife and old age.^9^

Most previous research in this field has examined whether depression is a risk factor for incident CHD or a risk factor for adverse cardiac outcomes among patients with CHD.^2–5^ ^22–24^ Relatively little is known about CHD or its risk factors as predictors of depression or as in our study, more general common mental disorder, as indicated by psychological distress. Our findings are in line with the evidence supporting manifest CHD to be associated with the risk of depression.^5^ Our findings also support previous studies which have shown the adverse symptom trajectories to be more common among women and individuals with low socioeconomic status.^9^ ^25^ Our approach to CHD and its risk factors using long-term trajectory modelling of psychological distress is new. In the two previous studies on depression, CHD was self-reported and only in one study hypertension and obesity were clinically measured.^10^ ^11^ Both these studies included participants who were elderly (>65 years) at baseline and followed for 20 years. The findings were inconsistent; in a study including only women, MI, hypertension and obesity predicted increasing and persistently high depressive symptom trajectories compared with persistently low,^10^ while another, smaller study of men and women did not find any associations.^11^

Our study adds new insight by using a relatively large dataset and clinically verified CHD in a middle-aged population. We found that participants with pre-existing CHD had a 1.9-fold increased risk of ending up on a persistently high and a 1.7-fold risk of intermediate to low, when compared with persistently low trajectory. There are at least two potential mechanisms that explain these results. According to the vascular depression hypothesis,^5^ ^6^ ^26^ long-term symptomatic trajectory may be the result of vascular pathology itself. However, in our cohort, smoking and obesity but not more direct indicators of vascular pathology, such as hypertension and hypercholesterolaemia, were associated with persistently high trajectory. Another viewpoint states that long-term trajectory of symptoms can be a component of prolonged adjustment disorder in response to a severe chronic disease and its impact on the quality of life.^3^ ^27^ The association found between prevalent CHD and the development from intermediate to low trajectory may reflect this type of relatively short-term adjustment disorder.

Of the CHD risk factors, obesity and smoking were associated with persistently intermediate and persistently high symptom trajectories. Behaviour-related factors have rarely been investigated in this context and the findings from the few studies available are inconsistent.^9^ ^11^ ^25^ Our results add evidence of the potential benefit of tackling obesity and smoking in the attempts to prevent long-lasting mental health problems. However, further intervention studies are needed to test whether these associations are causal. Further research should also examine the mechanisms, such as systemic inflammation, underlying the link between CHD, obesity, smoking and mental health trajectories. Although we found limited support for the vascular origin hypothesis, it is important to note that CHD and the risk factors were based on one-time assessments at baseline and we did not account for time-varying changes in them. They may change over time, for example, due to new events and new treatment practices. As lipid-lowering drugs became increasingly common over the follow-up, it might have diluted the association with hypercholesterolaemia.

A major strength of this study is a long-term follow-up of psychological distress with up to seven measurement points and a valid, clinically ascertained CHD at the baseline. Data on BMI, hypertension, hypercholesterolaemia and diabetes were based on clinical examinations. The study also has limitations. The GHQ-30 is a screening instrument, designed to capture symptoms of depression, anxiety, insomnia and social dysfunction rather than providing a clinical diagnosis of depressive disorders. However, it has been shown to have high validity for depression in the present cohort.^15^ For elderly cohorts, these types of non-specific instruments have been suggested to be more suitable than those assessing clinical diagnoses of depressive disorder because the manifest disease is often non-specific and multimorbid.^12^ Although this study provides a novel approach to research on long-term associations between CHD and psychological distress, a replication with a larger number of baseline CHD cases is needed.

Loss to follow-up may also have affected the trajectories estimated. In the present cohort, those who died had an average of four measurements while the mean number of measurements in the total cohort was six out of the maximum of seven. The mean number of measurements during follow-up was similar (6.0, p=0.520) among baseline distress cases and non-cases although the CHD cases had fewer (5.7) measurements than non-cases (6.0; p=0.003). Because the number of assessments was satisfactory in all groups, we believe that these differences are not likely to have introduced major bias. In addition, while GBTM identifies a certain number of discrete latent trajectories in observed data, it cannot prove that such discrete trajectories actually exist and remain as observed over time. GBTM also has difficulty in detecting subgroups that represent a very small portion of the population which, however, was not an issue in our study. Furthermore, factors that were not measured in our study, such as severity of disease, individual coping strategies^28^ and cardiac rehabilitation interventions among individuals with CHD^24^ may affect the mental health prognosis. Further studies should also examine whether the associations are bidirectional, that is, whether being on a long-term trajectory of symptoms predict incident CHD or whether a combination of long-term symptoms and CHD increases the risk of premature death.^29^ Finally, as the Whitehall II study is an occupational white-collar cohort, replications with general populations are also needed.

We have shown that over two-thirds of middle-aged participants followed trajectories through old age that were characterised by low probability of psychological distress while 7% were on a trajectory of persistent distress. This study is in line with the current guidelines which highlight the assessment of mental health among patients with CHD.^30^ Clinicians should be aware of the potential long-lasting associations of CHD, obesity and smoking as risk factors for prolonged psychological distress.

**Key messages**

**What is already known on this subject?**

- Coronary heart disease (CHD) and its risk factors have been found to predict symptoms of mental disorders, particularly depression.
- Their associations with long-term trajectories of mental ill health from midlife to old age remain unclear.

**What might this study add?**

- Trajectory analyses over 21 years showed that CHD was associated with a 1.9-fold, smoking with a 1.6-fold and obesity with a 1.5-fold likelihood of persistent psychological distress trajectory from midlife to old age.

**How might this impact on clinical practice?**

- Clinicians should be aware that persons who smoke, have CHD or are obese are at increased risk of having long-lasting psychological distress symptoms.
