# Supplementary material for: Coronary heart disease and risk factors as predictors of trajectories of psychological distress from midlife to old age
Source: Heart. 2016 Nov 18;103(9):659–65. doi: 10.1136/heartjnl-2016-310207 (PMC5529979; doi:10.1136/heartjnl-2016-310207)
Supplement: Supplementary table 1 [file heartjnl-2016-310207supp001.pdf]

Supplementary Table S1 Model comparisons for trajectories of psychological distress

	Fit indices	
Model*	BIC	AIC
Three-trajectory solutions		
1 1 1	-19930.42	-19902.69
1 1 2	-19914.60	-19883.41
1 2 1	-19927.04	-19895.84
1 2 2	-19916.98	-19882.31
2 1 1	-19931.03	-19899.84
2 1 2	-19914.93	-19880.26
2 2 1	-19917.88	-19883.22
2 2 2	-19911.17	-19873.04
Four-trajectory solutions		
1 1 1 1	-19897.53	-19859.40
1 1 1 2	-19880.06	-19838.47
1 1 2 1	-19877.34	-19835.74
1 2 1 1	-19901.35	-19859.76
1 1 2 2	-19866.64	-19821.58
1 2 1 2	-19883.83	-19838.77
1 2 2 1	-19876.95	-19831.89
1 2 2 2	-19865.08	-19816.55
2 2 2 2†	-19860.28	-19808.28
2 2 2 1	-19872.23	-19823.70
2 2 1 2	-19886.10	-19837.57
2 1 2 2	-19865.08	-19816.55
2 2 1 1	-19867.76	-19822.70
2 1 2 1	-19876.95	-19831.89
2 1 1 2	-19866.64	-19821.58
2 1 1 1	-19877.34	-19835.74

BIC (Bayesian information criterion), AIC (Akaike's information criterion).
*1=linear trajectory, 2=non-linear trajectory.
† Chosen model with the best fit.
