# Supplementary material for: Coronary heart disease and risk factors as predictors of trajectories of psychological distress from midlife to old age
Source: Heart. 2016 Nov 18;103(9):659–65. doi: 10.1136/heartjnl-2016-310207 (PMC5529979; doi:10.1136/heartjnl-2016-310207)
Supplement: Supplementary table 2 [file heartjnl-2016-310207supp002.pdf]

Supplementary Table S2 Multivariable adjusted multinomial logistic regression analysis for the associations of sociodemographic factors and cardiovascular risk factors at baseline with trajectories of psychological distress

	'Persistently intermediate' versus 'persistently low'	'Intermediate to low' versus 'persistently low'	'Persistently high' versus 'persistently low'
	Odds ratio (95% CI)*	Odds ratio (95% CI)*	Odds ratio (95% CI)*
Age: <50 years	1.00	1.00	1.00
≥50 years	0.82 (0.70-0.96)	0.41 (0.34-0.48)	0.55 (0.45-0.68)
Sex: male	1.0	1.00	1.00
Female	1.36 (1.14-1.62)	1.52 (1.26-1.82)	1.50 (1.19-1.88)
Socioeconomic status: high	1.00	1.00	1.00
Intermediate	1.00 (0.85-1.18)	1.04 (0.88-1.24)	1.38 (1.09-1.74)
Low	1.02 (0.79-1.32)	0.95 (0.72-1.26)	1.74 (1.25-2.43)
Smoking: no	1.00	1.00	1.00
Yes	1.33 (1.07-1.64)	1.16 (0.92-1.46)	1.55 (1.19-2.04)
High alcohol use: within recommended limits	1.00	1.00	1.00
No use	1.09 (0.89-1.33)	0.92 (0.74-1.15)	1.21 (0.93-1.56)
Above recommended limits	1.13 (0.92-1.39)	1.09 (0.88-1.35)	1.12 (0.84-1.49)
Obesity: no	1.00	1.00	1.00
Yes	1.33 (1.04-1.70)	1.09 (0.83-1.44)	1.47 (1.07-2.01)
Hypertension: no	1.00	1.00	1.00
Yes	1.02 (0.85-1.24)	0.94 (0.76-1.16)	1.00 (0.77-1.30)
High total cholesterol (≥5.0 mmol/L): no	1.00	1.00	1.00
Yes	1.25 (0.95-1.66)	1.01 (0.78-1.31)	1.09 (0.77-1.54)
Total cholesterol level (mmol/L): <5.0	1.00	1.00	1.00
5.0- <6.5	1.19 (0.89-1.59)	1.02 (0.78-1.33)	1.11 (0.78-1.59)
≥6.5	1.31 (0.98-1.76)	0.99 (0.75-1.30)	1.05 (0.73-1.51)
Diabetes: no	1.00	1.00	1.00
Yes	1.12 (0.71-1.75)	1.30 (0.79-2.12)	0.83 (0.41-1.70)

*All variables are mutually adjusted and additionally adjusted for coronary heart disease and history of psychological distress at baseline.
